# Meprin Metalloprotease Deficiency Associated with Higher Mortality Rates and More Severe Diabetic Kidney Injury in Mice with STZ-Induced Type 1 Diabetes

**DOI:** 10.1155/2017/9035038

**Published:** 2017-07-19

**Authors:** John E. Bylander, Faihaa Ahmed, Sabena M. Conley, Jean-Marie Mwiza, Elimelda Moige Ongeri

**Affiliations:** ^1^Department of Environmental Sciences, Pennsylvania State University, Harrisburg, Middletown, PA 17057, USA; ^2^Department of Biology, North Carolina A&T State University, Greensboro, NC 27411, USA

## Abstract

Meprins are membrane-bound and secreted metalloproteinases consisting of *α* and/or *β* subunits that are highly expressed in kidney epithelial cells and are differentially expressed in podocytes and leukocytes (macrophages and monocytes). Several studies have implicated meprins in the progression of diabetic nephropathy (DN) and fibrosis-associated kidney disease. However, the mechanisms by which meprins modulate DN are not understood. To delineate the role of meprins in DN, we subjected meprin *αβ* knockout (*αβ*KO) mice and their wild-type (WT) counterparts to streptozotocin-induced type 1 diabetes. The 18-week survival rates were significantly lower for diabetic meprin *αβ*KO mice when compared to those for their WT counterparts. There were significant decreases in mRNA and protein levels for both meprin *α* and *β* in diabetic WT kidneys. Furthermore, the blood urea nitrogen levels and urine albumin/creatinine ratios increased in diabetic meprin *αβ*KO but not in diabetic WT mice, indicating that meprins may be protective against diabetic kidney injury. The brush border membrane levels of villin, a meprin target, significantly decreased in diabetic WT but not in diabetic meprin *αβ*KO kidneys. In contrast, isoform-specific increases in cytosolic levels of the catalytic subunit of PKA, another meprin target, were demonstrated for both WT and meprin *αβ*KO kidneys.

## 1. Introduction

Meprins are zinc metalloproteases of the astacin family that are abundantly expressed in the brush border membranes (BBM) of kidney proximal tubules and small intestines [1, 2]. Other locations with documented meprin expression include the skin [3], podocytes [4], and leukocytes (macrophages and monocytes) [5]. Meprins are composed of two subunits, alpha (*α*) and beta (*β*), that are encoded by distinct genes on chromosomes 6 and 18, respectively, in humans, and chromosomes 17 and 18, respectively, in mice [6–8]. These result in two protein isoforms, meprin A and meprin B. Meprin A is a homooligomer of *α* subunits or a heterooligomer of *α* and *β* subunits, while meprin B is a homooligomer of *β* subunits [9, 10]. Meprins have been implicated in the pathology of acute and chronic kidney injury. The levels of expression and localization of meprins have been associated with acute kidney injury induced by ischemia/reperfusion (IR) [11, 12]. While meprin localization is restricted to the BBM in healthy kidneys, both meprin A and meprin B get redistributed to the cytoplasm and basolateral membranes in IR [11, 12]. Pretreatment of rats with the meprin inhibitor, actinonin, and disruption of the meprin *β* gene both protected mice from IR-induced renal injury [11, 13], suggesting that meprins enhance kidney injury in IR.

A role for meprin *β* in the progression of diabetic nephropathy (DN) and fibrosis-associated kidney disease has been demonstrated by several studies in both rodents and humans [14–16]. Db/db mice, a common mouse model of type 2 diabetes, manifested decreased meprin *α* and meprin *β* gene and protein expression before the development of clinical kidney disease [15]. Furthermore, meprin A enzymatic activity was lower in the diabetic db/db mice when compared to that in the control nonobese mice. Rats with streptozotocin- (STZ-) induced type 1 diabetes were also shown to have significant increases in urinary meprin A excretion. Additionally, their kidney proximal tubule BBM protein levels and meprin A activity were both decreased [15]. In humans, meprin *β* gene polymorphisms were associated with DN in the Pima Indians, a United States Native American ethnic group with an extremely high incidence of type 2 diabetes, and a prevalence of end stage renal disease (ESRD) that is 23 times that of the general population [16]. A study involving 154 diabetic individuals revealed 19 single-nucleotide polymorphisms (SNPs) of the meprin *β* gene. Nine of the SNPs had significant within-family associations, suggesting a role for meprin *β* in DN. However, the mechanisms by which meprins modulate the progression of DN are not known. Several extracellular (ECM) proteins (e.g., collagen IV, fibronectin, laminin, and nidogen-1) and modulators of inflammation (e.g., IL-1*β*, IL-6, IL-18, and MCP-1) are proteolytically processed by meprins, suggesting that meprins could impact inflammation and the fibrosis observed in DN. The objective of the present study was to evaluate how meprin deficiency impacts the pathology of kidney injury in mice with STZ-induced type 1 diabetes.

## 2. Materials and Methods

### 2.1. Experimental Animals

Eight-week-old male mice on a congenic C57BL/6 background were used for these studies. We compared wild-type (WT) and meprin *αβ* knockout (*αβ*KO) mice in which the genes for both meprin *α* and meprin *β* were disrupted. The mice were housed at the Pennsylvania State University, College of Medicine, Hershey, Pennsylvania. All the mice were kept under a 12 : 12 light: dark cycle and provided rodent chow and fresh deionized water ad libitum. The animal procedures used were approved by the Institutional Animal Care and Use Committees (IACUC).

### 2.2. Induction of Diabetes

Type 1 diabetes was induced in male mice at age 8 weeks (*n* = 7 for each group) by intraperitoneal injection of STZ. We first used high-dose STZ (100 mg/kg body weight for 2 days) to determine whether diabetes affects meprin expression. The WT mice for this experiment were sacrificed at 12 weeks post-STZ injection and real-time PCR was used to evaluate the expression of meprin *α* and meprin *β*. After confirming a decrease in meprin mRNA levels, we sought to find out how meprin expression impacts the progression of DN. To this end, we induced type 1 diabetes in both WT and meprin *αβ*KO mice. However, because of the side effects associated with high-dose STZ, we used low-dose STZ and planned to keep the mice for 24 weeks, a time point that has been used for C57BL/6 mice in previous studies. The mice were injected with low-dose STZ, (55 mg/kg daily for 5 consecutive days) following the protocol described by Tesch and Allen [17] and recommended by the Animal Models of Diabetic Complications Consortium (AMDCC; http://amdcc.org/). The mice were fasted for 6 hours before STZ injection. The STZ was dissolved in sodium citrate solution (pH 4.5) and injected within 15 minutes using insulin syringes. Control mice (*n* = 4 for each group) were injected with equivalent volumes of the sodium-citrate vehicle solution. Biochemical assessments of diabetes were done by measuring blood glucose levels at 10 days post-STZ injection. STZ-injected mice with a nonfasting glucose of >15 mmol/L (>280 mg/DL) were considered diabetic and used for the study. Body weights were monitored on a weekly basis. Blood and urine samples were collected at 4-week intervals. Blood was sampled into heparin tubes by tail nicking and by cardiac puncture or aspiration from the abdominal aorta at the time the mice were sacrificed. Spot urine samples were collected by bladder massage onto sterile petri dishes, and metabolic cages were used for collecting 24 h urine samples. The high mortality rate associated with meprin deficiency was unexpected, leading us to sacrifice the mice at 18 weeks post-STZ injection so we could analyze the kidney tissue and avoid losing all the meprin *αβ* KO mice.

### 2.3. Biochemical Assays for Kidney Function

Kidney function was evaluated by assays for blood urea nitrogen (BUN), serum creatinine, and urine albumin to creatinine ratio. Measurement of BUN was performed with Vitros DT60II chemistry slides (Ortho Clinical Diagnostics, Rochester, NY). Serum and urine creatinine were assayed by ELISA (Diazyme Laboratories, San Diego, CA). Urinary albumin excretion was also determined by ELISA (Exocell, Philadelphia PA).

### 2.4. Processing of Kidney Tissue

The mice were weighed and sacrificed by isoflurane asyphyxiation followed by cervical dislocation. The kidneys were excised, decapsulated, and individually weighed. One longitudinal half of the right kidney tissue was fixed in methyl Carnoy's fixative (6 : 3 : 1 methanol-chloroform-acetic acid), transferred to 70% ethanol, and subsequently paraffin embedded for histological analysis. The other half was combined with the left kidney and snap frozen in liquid nitrogen for protein extraction. The left kidneys from mice subjected to the high-dose STZ treatment were homogenized immediately in TRIzol and processed for RNA extraction.

### 2.5. RNA Extraction

Total RNA was extracted from the left kidneys of the mice injected with high-dose STZ using TRIzol (Invitrogen, Carlsbad, CA). The quantity and integrity of total RNA were determined using an absorption ratio at 260 and 280 nm and agarose-formaldehyde gel electrophoresis, respectively.

### 2.6. Real-Time PCR

Synthesis of cDNA was performed with 2 *μ*g of each RNA preparation, SuperScript III reverse transcriptase (Invitrogen), and hexanucleotide random primers (Roche, Indianapolis, IN). A reaction without reverse transcriptase was run in parallel for each RNA sample to control for DNA amplification. PCR primers were designed with Primer Express 1.5 software (Applied Biosystems), and the Spidey data-mining tool (National Center for Biotechnology Information) was used to minimize DNA amplification. Primers were as follows: meprin *α* forward, CGCCTCAAGTCTTGTGTGGATT; reverse, ATTTCATGTTCAATGGTGGCCTT (product size, 164 bp); meprin *β* forward, AGGATTCAGCCAGGCAAGGA; reverse, CGTGACGATGGTAGACTCTGTCC (product size, 144 bp); and *β*-actin forward, TGACGTTGACATCCGTAAAGACC; reverse, CTCAGGAGGAGCAATGATCTTGA (product size, 148 bp). Quantitative fluorescent real-time PCR analysis was performed in an ABI 7700 sequence detector (Applied Biosystems) using the QuantiTect SYBR green PCR kit and 300 nM gene-specific primers. The cycle profile was 15 min at 95°C followed by 40 cycles of 15 s at 94°C, 30 s at 55°C, and 30 s at 71°C. Analyses of 40 ng of cDNA for meprin *β* and 16 ng for meprin *α* and *β*-actin were performed in triplicate, and reverse transcriptase negative control reactions were performed in duplicate. For determination of standard curves and PCR efficiencies, standards were prepared using dilutions of C57Bl/6 mouse kidney total RNA. Differences between slopes were <0.1, and PCR efficiencies were >1.97 for all primer pairs. Data were normalized to *β*-actin and analyzed using the comparative threshold cycle method. The results are presented as fold expression relative to nondiabetic (sodium citrate-injected) control mice.

### 2.7. Kidney Protein Extraction

The frozen kidney tissues were thawed on ice and homogenized in 9 volumes of homogenization buffer (2 mM Tris-HCl and 10 mM Mannitol; pH 7.0) with protease inhibitors and fractionated into BBM- and cytosolic-enriched protein fractions as previously described [12, 18]. In addition, RIPA buffer was used to obtain nonfractionated total protein lysates from each kidney. The protein levels were quantified using a Bradford protein assay and Bio-Rad's protein reagent and subsequently stored in aliquots at −80°C until analyzed by Western blot.

### 2.8. Western Blot Analysis

Western blot analysis coupled with optic densitometry were used to quantify the protein levels for meprin A, meprin B, villin, the catalytic subunit of protein kinase A (PKA C), and phosphor-PKA C in kidney tissue. To this end, 20 *μ*g of BBM-enriched proteins or 60 *μ*g of cytosolic-enriched protein samples were electrophoretically separated using 10% acrylamide gels and transferred onto nitrocellulose membranes. Nonspecific binding sites on the membranes were blocked in 5% fat-free milk made in tris-buffered saline with 0.5% Tween 20 (TBS-T) for 1 h at room temperature (RT). The membranes were then probed using primary antibodies against each of the proteins and incubated at RT for 1 h or at 4°C overnight. The antibodies used were rabbit polyclonal antimeprin *α* (HMC14, gift from Dr. Judith Bond, Pennsylvania State University, College of Medicine, Hershey, Pennsylvania) diluted 1 : 3000, rabbit polyclonal antimeprin *β* (HMC77, gift from Dr. Judith Bond, Pennsylvania State University, College of Medicine, Hershey, Pennsylvania) diluted 1 : 5000, rabbit polyclonal antivillin (Santa Cruz Biotechnology, Santa Cruz CA) diluted 1 : 1000, mouse monoclonal anti-PKA C (BD Biosciences, San Jose, CA) diluted 1 : 1000, and phosphor-PKA C (Cell Signaling, Danvers, MA) diluted 1 : 1000. The nitrocellulose membranes were washed three times for 10 min in TBS-T and incubated with HRP-conjugated secondary antibodies (Bio-Rad, Hercules, CA) diluted 1 : 15,000 in TBS-T at RT for 1 h or at 4°C overnight. The membranes were then washed in TBS-T for 15 min, three times, and exposed to Chemiluminiscence substrate, and protein bands developed by exposure to X-ray film. The membranes were stripped and probed for tubulin as a loading control. The intensities of the protein bands were quantified using Bio-Rad's GS800 calibrated densitometer coupled with QuantityOne software. The relative OD were calculated as a ratio of the OD for each protein and the OD for the tubulin loading control. The relative OD data were subjected to statistical analysis using 2-way ANOVA (GraphPad Prism software).

### 2.9. Immunohistochemical Analysis and Microscopic Imaging

Kidney tissues were paraffin embedded at the Pennsylvania State University, College of Medicine, Histology Core Facility, Hershey, PA. Serial kidney sections (4 *μ*m) were cut onto Superfrost® slides and deparaffinized by sequential exposure to xylenes (3 × 5 minutes), 100% ethanol (2 × 10 minutes), 95% ethanol (3 × 5 minutes), and distilled water (2 × 5 minutes). The kidney tissue sections were stained in periodic acid-Schiff and hematoxylin-eosin staining to evaluate tubular dilation. Analysis for localization of meprins was performed using standard immunohistochemical protocols previously described [12, 19]. The sections were imaged using light microscopy and ImageJ was used to evaluate tubular dilation.

### 2.10. Statistical Analysis

The ELISA assays were performed in triplicate and analyzed by two-way ANOVA. The intensity of protein bands for Western blot were quantified using Bio-Rad's GS800 calibrated densitometer and QuantityOne software (Bio-Rad, Hercules, CA). The densitometric data for Western blots for individual mouse samples were normalized to tubulin levels and analyzed by two-way ANOVA with pairwise comparisons for all treatment groups using GraphPad Prism software. The data are presented as mean ± SEM. *p* values ≤0.05 were considered statistically significant. Comparison of survival curves utilized chi-square and the Gehan-Breslow-Wilcoxon test.

## 3. Results

### 3.1. Severe Diabetes Caused a Significant Decrease in Kidney Meprin mRNA and Protein Levels

**Figure 1:**
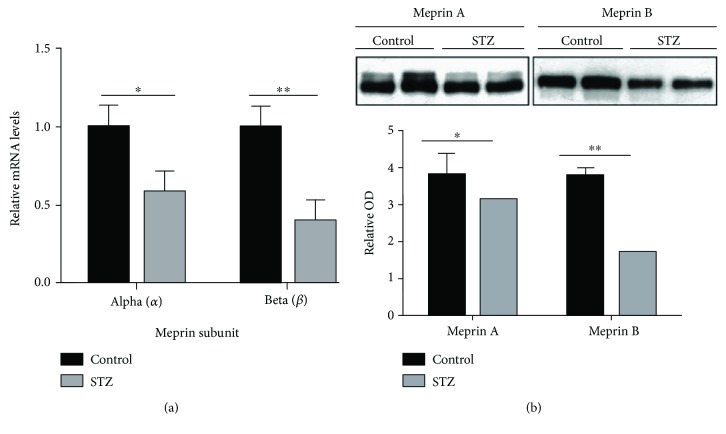
(a) Messenger RNA levels for meprin *α* and meprin *β*. Kidneys from WT mice injected with high-dose STZ were harvested at 12 weeks post-STZ injection and Trizol was used to extract RNA. Real-time PCR was used to amplify meprin *α* and meprin *β* mRNA. Data were normalized to *β*-actin and analyzed using the comparative threshold cycle method. The results are presented as fold expression ± SEM (*n* = 7) relative to nondiabetic (sodium citrate-injected) control mice. ^∗^ *p* < 0.05, ^∗∗^ *p* < 0.01. (b) Protein levels for meprin A and meprin B in the brush border membrane (BBM) of kidneys from wild-type (WT) mice with STZ-induced type 1 diabetes. Proteins were extracted from WT mice injected with low-dose STZ at 18 weeks post STZ injection, and Western blot analysis used to evaluate meprin protein levels in the BBM-enriched fraction. Diabetic kidneys had a significant decrease in the levels of both meprin A and meprin B proteins (*p* ≤ 0.05). However, the fold change in protein levels was greater for meprin B (53%) than for meprin A (18%). ^∗^ *p* < 0.05, ^∗∗^ *p* < 0.01.

We first determined the impact of severe diabetes on renal meprin expression levels. Wild-type mice treated with high-dose STZ were sacrificed at 12 weeks post-STZ injection, and real-time PCR was used to determine the kidney meprin *α* and meprin *β* mRNA levels. The diabetic mice had a significant decrease in kidney mRNA levels of 43% for meprin *α* (*p* ≤ 0.05) and 61% for meprin *β* (*p* ≤ 0.01) (Figure 1(a)). Western blot analysis with BBM-enriched kidney protein fractions also demonstrated an 18% decrease in BBM meprin *α* protein levels (*p* ≤ 0.05) and a 53% decrease in meprin *β* protein levels (*p* ≤ 0.01) in mice injected with low-dose STZ at 18 weeks post-STZ (Figure 1(b)).

### 3.2. Meprin Deficiency Was Associated with Higher Mortality Rates in Diabetic Mice

**Figure 2:**
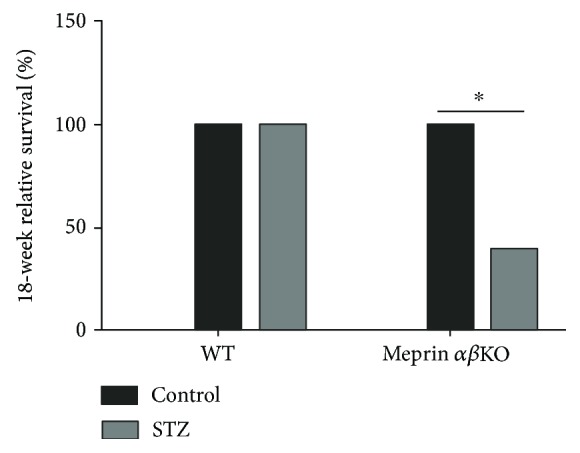
Survival rates for mice with STZ-induced type 1 diabetes. Low-dose STZ was used to induce type 1 diabetes in 8-week-old male mice. The relative survival rates at 18 weeks post-STZ were normalized to those of nondiabetic control mice in each group. Meprin *αβ* double-KO mice had a significantly lower 18-week survival rate (40%) when compared to wild-type mice (100%) (*p* ≤ 0.02). ^∗^ *p* ≤ 0.05.

We initially sought to evaluate the impact of meprin deficiency on the pathology of DN over a 24-week period. To this end, we used low-dose STZ to induce type 1 diabetes. The meprin *αβ*KO mice (with disruption of both meprin *α* and meprin *β* genes) with STZ-induced type 1 diabetes had significantly lower relative survival rates at 18 weeks compared to wild-type mice (*p* ≤ 0.02) (Figure 2). The 18-week survival rate for the meprin *αβ*KO mice was only 40%, leading us to terminate the experiment at 18 weeks post-STZ injection. The relative survival rates for each group were determined as a ratio of the survival of the diabetic mice to that of the nondiabetic control mice in the same genotype.

### 3.3. Meprin Deficiency Resulted in Greater Impairment of Kidney Function

**Figure 3:**
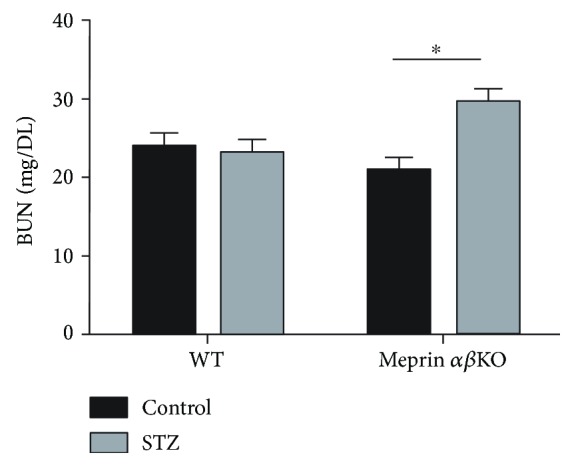
Blood urea nitrogen (BUN) levels at 12 weeks post-STZ injection. Blood was collected by tail nicking and plasma BUN levels were determined by use of BUN slides that were read on a Vitros DT6011 analyzer (Ortho Clinical Diagnostics, Rochester, NY). BUN levels were significantly higher (*p* ≤ 0.05) in diabetic meprin *αβ* double-KO mice when compared to those in nondiabetic controls at 12 weeks post-STZ injection. This difference was not significant in the wild-type mice. ^∗^ *p* ≤ 0.05.

**Figure 4:**
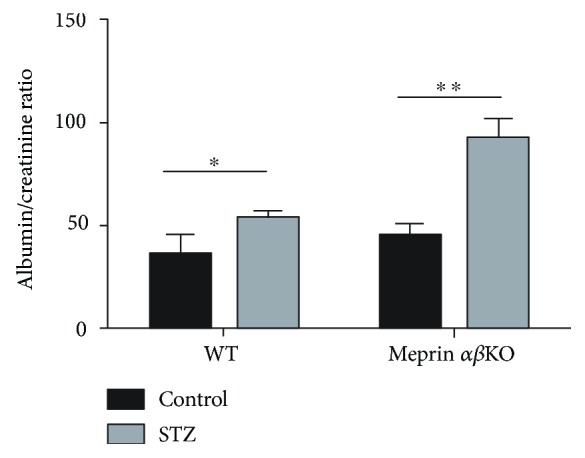
Urine albumin/creatinine ratio. Urine samples were collected onto sterile petri dishes by bladder massage. Albumin levels were determined by ELISA using murine Albuwell ELISA kits (Exocell, Philadelphia, PA). Creatinine levels in the same samples were determined by use of ELISA (Diazyme Laboratories). The 8-week albumin/creatinine ratios were significantly higher in diabetic meprin *αβ*KO mice when compared to those in their diabetic WT counterparts (*p* ≤ 0.05). ^∗^ *p* ≤ 0.05, ^∗∗^ *p* ≤ 0.01.

**Figure 5:**
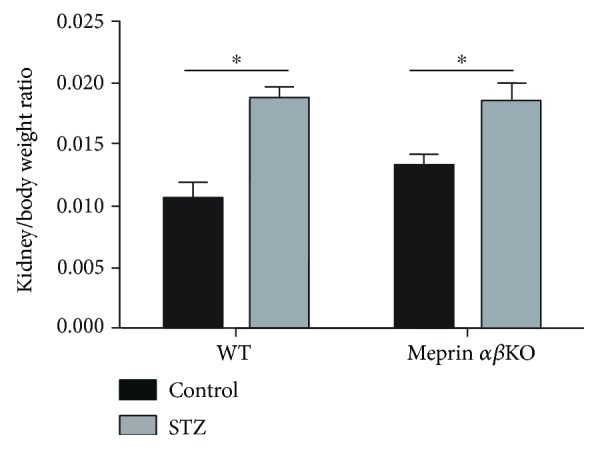
The kidney/body weight ratio at 18 weeks post-STZ injection. Kidneys were decapsulated and individually weighed. There was a significant renal hypertrophy in diabetic mice when compared to that in nondiabetic control mice in both genotypes. However, there was no significant difference between meprin *αβ*KO mice and WT mice. ^∗^ *p* ≤ 0.05.

Kidney phenotyping data showed greater loss of kidney function in meprin-deficient mice. The meprin *αβ*KO mice with low-dose STZ-induced type 1diabetes had progressively higher BUN levels at 4 and 8 weeks post-STZ injection. At 12 weeks post-STZ, the BUN levels were significantly higher (*p* ≤ 0.05) in diabetic meprin *αβ*KO mice when compared to those in their WT counterparts (Figure 3). Another important finding was a significantly higher albumin/creatinine ratio in diabetic meprin *αβ*KO mice when compared to that in their diabetic WT counterparts at 8 weeks post-STZ (Figure 4). While renal hypertrophy was significantly higher in diabetic kidneys, there was no significant association with meprin expression or deficiency (Figure 5).

### 3.4. Levels and Phosphorylation Status of the Catalytic Subunit of Protein Kinase A (PKA C) Correlated with Meprin Expression in Diabetic Kidneys

**Figure 6:**
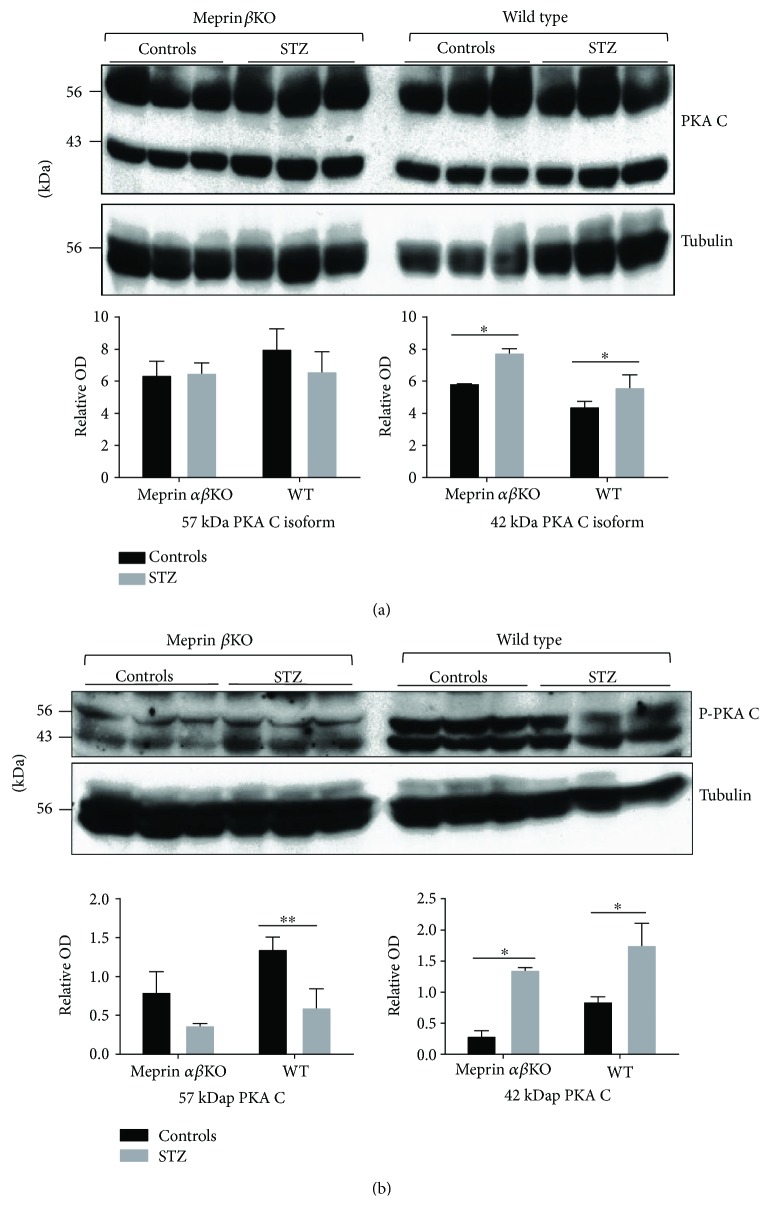
(a) Representative immunoblot for the catalytic subunit of protein kinase A (PKA C) in cytosolic kidney protein fraction; (b) representative immunoblot for p-PKA C in cytosolic-enriched kidney proteins. Proteins were extracted from kidney tissue at 18 weeks post-STZ injection and fractionated into cytosolic- and BBM-enriched fractions using differential centrifugation. Western blot analysis using anti-PKA C and p-PKA C-specific antibodies coupled with optic densitometry were used to quantify the PKA C protein levels. The levels of the 42 kDa PKA C protein isoform (a) were significantly higher in kidneys from diabetic mice, but there were no significant changes in the levels of the 57 kDa isoform. The levels of the 42 kDa p-PKA C increased while the levels of the 57 p-KCA isoform decreased for both genotypes. ^∗^ *p* ≤ 0.05, ^∗∗^ *p* ≤ 0.01.

The PKA pathway has previously been shown to play a role in extracellular matrix (ECM) protein metabolism and the fibrosis associated with DN. Previous in vitro studies by our group had shown that the catalytic subunit of PKA (PKA C) is a meprin substrate and that meprin A is expressed in the glomeruli of diabetic mouse kidneys [19–21]. We therefore sought to evaluate the levels of phospho-PKA C in the kidneys of wild-type and meprin *αβ*KO mice with STZ-induced type 1 diabetes and compare them to those of nondiabetic controls for each genotype. Western blot analysis showed that the levels of the 42 kDa PKA C protein isoform in cytosolic-enriched kidney proteins were significantly higher in the kidneys from diabetic mice for both genotypes (Figure 6(a)). This increase was not observed for the 57 kDa PKA C isoform. We then determined the levels of phosphor-PKA C (p-PKA C) in the kidneys of diabetic mice. Western blot with phosphospecific anti-PKA C antibodies showed that the levels of the 42 kDa phospho-PKA increased in the kidneys of both WT and meprin *αβ*KO mice with STZ-induced diabetes when compared to those of their control counterparts. In contrast, the levels of the 57 kDa p-PKA C isoform decreased in both genotypes.

### 3.5. Diabetic Nephropathy Associated with a Decrease in Brush Border Villin Levels in Meprin Expressing Kidneys

**Figure 7:**
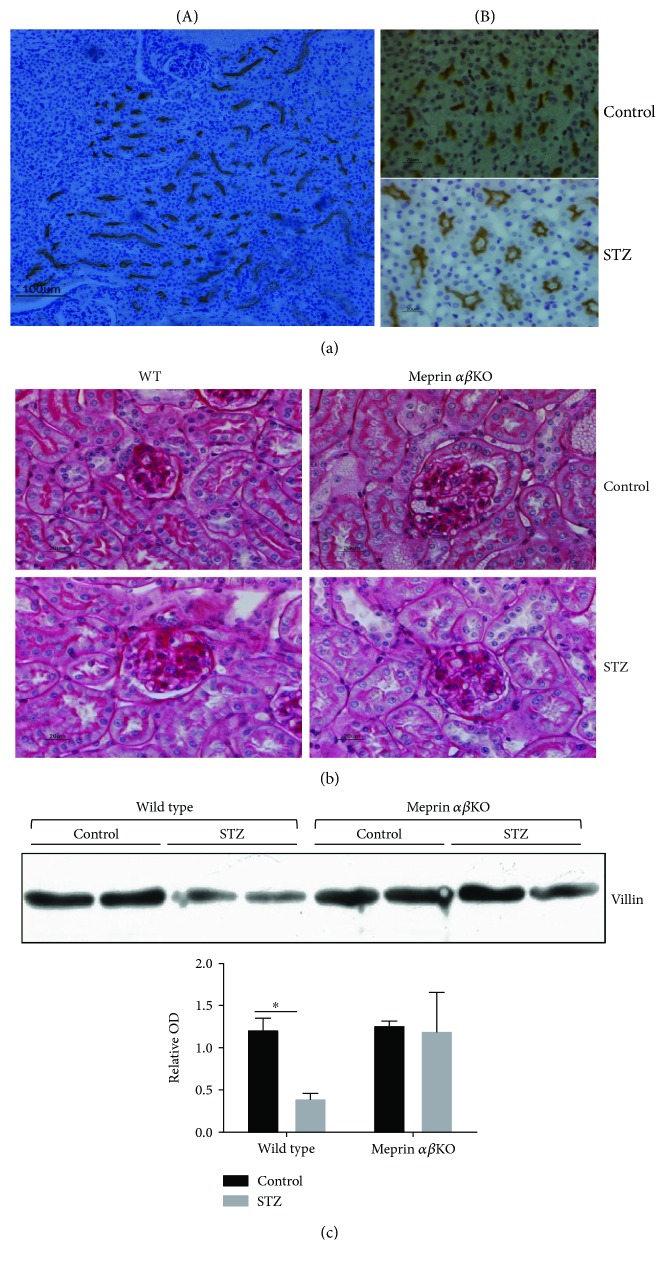
Localization of meprin B in the juxtamedullary region of the kidney (a), acid Schiff-stained kidney sections (b), and representative immunoblot for villin in BBM-enriched kidney protein fractions from wild-type and meprin *αβ*KO mice at 18 weeks post-STZ (c). There was no noticeable redistribution of meprins from the BBMs to other cell compartments. Significant tubular dilation was evident in the kidneys from diabetic mice when compared to that from nondiabetic controls. There was more tubular dilation in STZ-treated mice when compared to that in nondiabetic vehicle-treated controls. The levels of villin were significantly lower in diabetic WT mice when compared to those in nondiabetic controls (*p* ≤ 0.05). This decrease was not observed in meprin *αβ*KO mice. ^∗^ *p* ≤ 0.05.

Histological analysis showed loss of brush border microvilli and dilation of proximal tubules from the kidneys of diabetic wild-type mice (Figures 7(a) and 7(b)). This was especially apparent in stained sections probed with antimeprin antibodies (Figure 7(a)). To determine whether the tubular dilation is associated with loss of cytoskeletal proteins that constitute the structure of the brush border microvilli, we used Western blot analysis to compare the levels of villin, a meprin B target and a major component of the kidney proximal tubule BBM [12], in the BBM-enriched kidney proteins. Our data showed a significant decrease (*p* ≤ 0.05) in BBM villin levels in diabetic WT mice when compared to those in their meprin *αβ*KO counterparts (Figure 7(c)).

## 4. Discussion

Meprin metalloproteases have been implicated in the pathology of diabetic nephropathy (DN), the major single cause of end-stage renal disease (ESRD). Diabetic nephropathy is characterized by albuminuria [22], renal hypertrophy [23], and excess accumulation of extracellular matrix (ECM) within the glomerulus and interstitium [24–28]. In humans, polymorphisms in the meprin *β* gene were associated with DN in the Pima Indians of Arizona, a US ethnic group with an extremely high incidence of type 2 diabetes and ESRD [16]. Several studies have also demonstrated an association between meprin expression and the development of renal fibrosis, but the underlying mechanisms are not understood. Gene and protein expression levels for both meprin *α* and meprin *β* were downregulated in the kidneys of diabetic rats and in db/db mice before the development of overt kidney disease [15]. Data from the present study show a significant downregulation of gene and protein expression levels for both meprin *α* and meprin *β* in a mouse model of type I diabetes. By comparing congenic wild-type and meprin *αβ* knockout mice, we have shown more severe diabetic kidney damage in meprin-deficient mice as evidenced by significantly higher blood urea nitrogen (BUN) levels and higher albumin/creatinine ratios. Mice with disruption of the meprin *α* and meprin *β* genes also had higher mortality rates associated with STZ-induced type 1 diabetes.

Two key pathological changes in DN, thickening of the glomerular basement membrane (GBM) and tubular interstitial fibrosis, are directly related to excessive ECM protein buildup. Identified meprin targets in the kidney include ECM proteins and proteins involved in ECM metabolism and thus linked to the renal fibrosis associated with DN [12, 19, 29–32]. In vitro and in vivo studies showed that meprins proteolytically process several ECM proteins (e.g., type IV collagen, type VI collagen, nidogen-1, laminin, and fibronectin) [33–37] and could thus directly impact the levels of ECM proteins via degradation. Other meprin substrates involved in ECM metabolism include proinflammatory cytokines and chemokines (e.g., interleukin 1*β* (IL-1*β*) [31, 32], interleukin 6 (IL-6) [38], and prointerleukin-18 (pro-IL-18) [30] and chemoattractant protein-1 (MCP-1), the catalytic subunit of protein kinase A (PKA) [19, 39], and protein kinase C (PKC) [40]). Meprins were previously implicated in inflammation and fibrosis in other pathologies, for example, meprin *β* expression is downregulated in collagen IVA3 knockout mice that develop Alport's syndrome [41] and meprin *α* was identified as a susceptibility gene for inflammatory bowel disease (IBD) [42, 43]. The PKA signaling pathway has been shown to interact with TGF *β*1 signaling to regulate ECM metabolism. We recently demonstrated isoform-specific proteolytic processing of the catalytic subunit of PKA (PKAC) by meprins which resulted in reduced kinase activity [19]. We further showed meprin expression in the glomerular of mice with STZ-induced type 1 diabetes. The protein kinase A (PKA) signaling pathway has been shown to play a role in ECM metabolism. Meprins could thus modulate the progression of DN via modulation of ECM metabolism through interactions with the PKA signaling pathway. In the present study, we demonstrated modest increases in the cytosolic levels of the 42 kDa (corresponding to the size of PKA C*α* and C*β*1 splice variants) and 57 KDa PKA C (corresponding to the size of PKA C*β*4 splice variant) isoforms in diabetic nephropathy in both wild-type and meprin knockout mice. More importantly, we show a correlation between meprin expression and the phosphorylation status of PKA C isoforms. Since proteolytic processing by meprins reduces the kinase activity of PKA C, this could impact downstream mediators of the PKA signaling pathway in the kidney. The data suggest that in addition to directly cleaving ECM proteins, meprins could play an indirect role in ECM metabolism via proteolysis of signaling molecules such as PKA C, which are involved in ECM metabolism and the inflammatory response. Previous studies had shown that high blood glucose, the hallmark of diabetes, causes oxidative stress which induces synthesis of ECM proteins such as laminin and fibronectin in mesangial cells [44–46]. The glucose-mediated oxidative stress could be mimicked by the PKA agonist, forskolin, and inhibited by the PKA inhibitor, H89 [47]. Additionally, high glucose levels were shown to activate PKA, leading to phosphorylation and inhibition of G6PD in renal cortical extracts from diabetic rats [48]. Phosphorylation of the cAMP response-binding protein (CREB) also increased fibronectin production in rats with STZ-induced DN [45, 49]. The PKA signaling pathway further regulates TGF-*β*1 induction of epithelial-to-mesenchymal transition (EMT), an event that is related to the pathology of fibrotic kidney disease [50–52]. It is probable that the loss of kinase activity could impact downstream targets of the PKA signaling pathway, including TGF-*β*1-mediated regulation of the metabolism and catabolism of ECM, as well as the production of inflammatory response mediators. The downstream modulators of the PKA-TGF-*β*1-induced response are known to be cell type specific and depend on the cellular environment, and further studies are needed to delineate the impact of meprins on these pathways.

Meprins also cleave two cytoskeletal proteins, villin and actin, which would cause injury to the brush border membranes of proximal kidney tubules [12]. Data from the current study shows a significant reduction in the levels of villin in the kidneys from WT but not from meprin *αβ*KO mice. Villin is a critical part of the proximal tubule brush border membrane and degradation of villin could contribute to the appearance of dilation of the tubular lumen as observed in DN. While expression of meprins is most abundant in the BBM of proximal kidney tubules, meprins are redistributed from the BBM to the cytosol and basolateral membranes under pathological conditions such as ischemia reperfusion- (IR-) induced renal injury [11, 12]. Recent studies have documented the expression of meprins in rat podocytes [4] and in human and mouse leukocytes [5]. Our group recently demonstrated glomerular expression of meprins in the kidneys of diabetic mice but not in nondiabetic controls [19], suggesting that meprins could play a role in both tubular and glomerular renal pathology. Data from the present study indicate that meprin deficiency contributes to a more severe pathology observed in DN. Taken together, the data suggest that meprins play a protective role in kidney injury induced by diabetes. This could be via modulation of inflammation, an underlying cause of fibrosis, or through proteolytic processing of ECM proteins. Proteolytic processing by meprin B was shown to inactivate IL-6 [38]. In contrast, cleavage of IL-1*β* and pro-IL-18 by meprin B caused their activation [30, 31]. Meprin A was also shown to play a role in the release of the anti-inflammatory peptide, Ac-SDKP, from thymosin *β*4 [53]. Compared to human diabetic patients, rodent models of DN develop modest elevations in albuminuria and serum creatinine, and kidney histological lesions associated with DN in mice are also modest. This resistance to renal damage makes most strains of mice unsuitable as models for human DN. Because expression of meprins is much higher in the mouse kidneys when compared to that in humans [54], these data suggest that meprin expression may in part explain the modest DN observed in many mouse models of DN. Meprin-deficient mice could thus be a good model for studies on DN.

## References

[1] Kaushal G. P., Walker P. D., Shah S. V. (1994). An old enzyme with a new function: purification and characterization of a distinct matrix-degrading metalloproteinase in rat kidney cortex and its identification as meprin.

[2] Beynon R. J., Bond J. S. (1985). Expression of the Mep-1 gene regulating meprin, a kidney brush border proteinase.

[3] Becker-Pauly C., Howel M., Walker T. (2007). The alpha and beta subunits of the metalloprotease meprin are expressed in separate layers of human epidermis, revealing different functions in keratinocyte proliferation and differentiation.

[4] Oneda B., Lods N., Lottaz D. (2008). Metalloprotease meprin beta in rat kidney: glomerular localization and differential expression in glomerulonephritis.

[5] Sun Q., Jin H. J., Bond J. S. (2009). Disruption of the meprin alpha and beta genes in mice alters homeostasis of monocytes and natural killer cells.

[6] Bond J. S., Rojas K., Overhauser J., Zoghbi H. Y., Jiang W. (1995). The structural genes, MEP1A and MEP1B, for the alpha and beta subunits of the metalloendopeptidase meprin map to human chromosomes 6p and 18q, respectively.

[7] Jiang W., Dewald G., Brundage E. (1995). Fine mapping of MEP1A, the gene encoding the alpha subunit of the metalloendopeptidase meprin, to human chromosome 6P21.

[8] Reckelhoff J. F., Butler P. E., Bond J. S., Beynon R. J., Passmore H. C. (1988). Mep-1, the gene regulating meprin activity, maps between Pgk-2 and Ce-2 on mouse chromosome 17.

[9] Gorbea C. M., Marchand P., Jiang W. (1993). Cloning, expression, and chromosomal localization of the mouse meprin beta subunit.

[10] Gorbea C. M., Flannery A. V., Bond J. S. (1991). Homo- and heterotetrameric forms of the membrane-bound metalloendopeptidases meprin A and B.

[11] Bylander J., Li Q., Ramesh G., Zhang B., Reeves W. B., Bond J. S. (2008). Targeted disruption of the meprin metalloproteinase beta gene protects against renal ischemia-reperfusion injury in mice.

[12] Ongeri E. M., Anyanwu O., Reeves W. B., Bond J. S. (2011). Villin and actin in the mouse kidney brush-border membrane bind to and are degraded by meprins, an interaction that contributes to injury in ischemia-reperfusion.

[13] Carmago S., Shah S. V., Walker P. D. (2002). Meprin, a brush-border enzyme, plays an important role in hypoxic/ischemic acute renal tubular injury in rats.

[14] Sadlier D. M., Connolly S. B., Kieran N. E. (2004). Sequential extracellular matrix-focused and baited-global cluster analysis of serial transcriptomic profiles identifies candidate modulators of renal tubulointerstitial fibrosis in murine Adriamycin-induced nephropathy.

[15] Mathew R., Futterweit S., Valderrama E. (2005). Meprin-alpha in chronic diabetic nephropathy: interaction with the renin-angiotensin axis.

[16] Red Eagle A. R., Hanson R. L., Jiang W. (2005). Meprin beta metalloprotease gene polymorphisms associated with diabetic nephropathy in the Pima Indians.

[17] Tesch G. H., Allen T. J. (2007). Rodent models of streptozotocin-induced diabetic nephropathy.

[18] Kenny A. J., Ingram J. (1987). Proteins of the kidney microvillar membrane. Purification and properties of the phosphoramidon-insensitive endopeptidase (‘endopeptidase-2’) from rat kidney.

[19] Niyitegeka J. M., Bastidas A. C., Newman R. H., Taylor S. S., Ongeri E. M. (2015). Isoform-specific interactions between meprin metalloproteases and the catalytic subunit of protein kinase A: significance in acute and chronic kidney injury.

[20] Alhanaty E., Patinkin J., Tauber-Finkelstein M., Shaltiel S. (1981). Degradative inactivation of cyclic AMP-dependent protein kinase by a membranal proteinase is restricted to the free catalytic subunit in its native conformation.

[21] Alhanaty E., Shaltiel S. (1979). Limited proteolysis of the catalytic subunit of cAMP-dependent protein kinase—a membranal regulatory device?.

[22] Fioretto P., Caramori M. L., Dalla Vestra M., Mauer M. (2001). Risk predictors in patients with diabetic nephropathy.

[23] Duffy P. G., Johnston S. R., Donaldson R. A. (1984). Idiopathic retroperitoneal fibrosis in twins.

[24] Osterby R. (1973). Kidney structural abnormalities in early diabetes.

[25] Mauer S. M., Steffes M. W., Ellis E. N., Sutherland D. E., Brown D. M., Goetz F. C. (1984). Structural-functional relationships in diabetic nephropathy.

[26] Steffes M. W., Osterby R., Chavers B., Mauer S. M. (1989). Mesangial expansion as a central mechanism for loss of kidney function in diabetic patients.

[27] Steffes M. W., Bilous R. W., Sutherland D. E., Mauer S. M. (1992). Cell and matrix components of the glomerular mesangium in type I diabetes.

[28] Lane P. H., Steffes M. W., Fioretto P., Mauer S. M. (1993). Renal interstitial expansion in insulin-dependent diabetes mellitus.

[29] Jefferson T., Auf dem Keller U., Bellac C. (2013). The substrate degradome of meprin metalloproteases reveals an unexpected proteolytic link between meprin beta and ADAM10.

[30] Banerjee S., Bond J. S. (2008). Prointerleukin-18 is activated by meprin beta in vitro and in vivo in intestinal inflammation.

[31] Herzog C., Kaushal G. P., Haun R. S. (2005). Generation of biologically active interleukin-1beta by meprin B.

[32] Herzog C., Haun R. S., Kaushal V., Mayeux P. R., Shah S. V., Kaushal G. P. (2009). Meprin A and meprin alpha generate biologically functional IL-1beta from pro-IL-1beta.

[33] Kohler D., Kruse M., Stocker W., Sterchi E. E. (2000). Heterologously overexpressed, affinity-purified human meprin alpha is functionally active and cleaves components of the basement membrane in vitro.

[34] Kruse M. N., Becker C., Lottaz D. (2004). Human meprin alpha and beta homo-oligomers: cleavage of basement membrane proteins and sensitivity to metalloprotease inhibitors.

[35] Herzog C., Marisiddaiah R., Haun R. S., Kaushal G. P. (2015). Basement membrane protein nidogen-1 is a target of meprin beta in cisplatin nephrotoxicity.

[36] Walker P. D., Kaushal G. P., Shah S. V. (1998). Meprin A, the major matrix degrading enzyme in renal tubules, produces a novel nidogen fragment in vitro and in vivo.

[37] Kronenberg D., Bruns B. C., Moali C. (2010). Processing of procollagen III by meprins: new players in extracellular matrix assembly?.

[38] Keiffer T. R., Bond J. S. (2014). Meprin metalloproteases inactivate interleukin 6.

[39] Chestukhin A., Muradov K., Litovchick L., Shaltiel S. (1996). The cleavage of protein kinase A by the kinase-splitting membranal proteinase is reproduced by meprin beta.

[40] Boyd S., Newman R., Ongeri E. (2014). Protein kinase C alpha is a target for the meprin B metalloproteinase (690.14).

[41] Sampson N. S., Ryan S. T., Enke D. A., Cosgrove D., Koteliansky V., Gotwals P. (2001). Global gene expression analysis reveals a role for the alpha 1 integrin in renal pathogenesis.

[42] Banerjee S., Oneda B., Yap L. M. (2009). MEP1A allele for meprin A metalloprotease is a susceptibility gene for inflammatory bowel disease.

[43] Banerjee S., Jin G., Bradley S. G. (2011). Balance of meprin A and B in mice affects the progression of experimental inflammatory bowel disease.

[44] Singh L. P., Crook E. D. (2000). Hexosamine regulation of glucose-mediated laminin synthesis in mesangial cells involves protein kinases A and C.

[45] Singh L. P., Andy J., Anyamale V., Greene K., Alexander M., Crook E. D. (2001). Hexosamine-induced fibronectin protein synthesis in mesangial cells is associated with increases in cAMP responsive element binding (CREB) phosphorylation and nuclear CREB: the involvement of protein kinases A and C.

[46] Kreisberg J. I., Kreisberg S. H. (1995). High glucose activates protein kinase C and stimulates fibronectin gene expression by enhancing a cAMP response element.

[47] Hui L., Hong Y., Jingjing Z., Yuan H., Qi C., Nong Z. (2010). HGF suppresses high glucose-mediated oxidative stress in mesangial cells by activation of PKG and inhibition of PKA.

[48] Xu Y., Osborne B. W., Stanton R. C. (2005). Diabetes causes inhibition of glucose-6-phosphate dehydrogenase via activation of PKA, which contributes to oxidative stress in rat kidney cortex.

[49] Wang J., Huang H., Liu P. (2006). Inhibition of phosphorylation of p38 MAPK involved in the protection of nephropathy by emodin in diabetic rats.

[50] Wang L., Zhu Y., Sharma K. (1998). Transforming growth factor-beta1 stimulates protein kinase A in mesangial cells.

[51] Yang Y., Pan X., Lei W. (2006). Regulation of transforming growth factor-beta 1-induced apoptosis and epithelial-to-mesenchymal transition by protein kinase A and signal transducers and activators of transcription 3.

[52] Bu L., Qu S., Gao X. (2011). Enhanced angiotensin-converting enzyme 2 attenuates angiotensin II-induced collagen production via AT1 receptor-phosphoinositide 3-kinase-Akt pathway.

[53] Kumar N., Nakagawa P., Janic B. (2016). The anti-inflammatory peptide Ac-SDKP is released from thymosin-beta4 by renal meprin-alpha and prolyl oligopeptidase.

[54] Yura R. E., Bradley S. G., Ramesh G., Reeves W. B., Bond J. S. (2009). Meprin A metalloproteases enhance renal damage and bladder inflammation after LPS challenge.

