# Erratum to “Meprin Metalloprotease Deficiency Associated with Higher Mortality Rates and More Severe Diabetic Kidney Injury in Mice with STZ-Induced Type 1 Diabetes”

**DOI:** 10.1155/2018/2462697

**Published:** 2018-07-05

**Authors:** John E. Bylander, Faihaa Ahmed, Sabena M. Conley, Jean-Marie Mwiza, Elimelda Moige

**Affiliations:** ^1^Department of Environmental Sciences, Pennsylvania State University, Harrisburg, Middletown, PA 17057, USA; ^2^Department of Biology, North Carolina A&T State University, Greensboro, NC 27411, USA

In the article titled “Meprin Metalloprotease Deficiency Associated with Higher Mortality Rates and More Severe Diabetic Kidney Injury in Mice with STZ-Induced Type 1 Diabetes” [1], the meprins in Figure 1(b) were mistakenly duplicated due to a production error. The figure has been corrected in place.
